# Multifactorial drivers of engagement in sex work among Ethiopian women: a multinomial logistic regression approach

**DOI:** 10.3389/fgwh.2025.1512560

**Published:** 2025-07-14

**Authors:** Dawit Sekata Duressa, Lemessa Negeri Debel, Saro Abdella Abrahim

**Affiliations:** Department of HIV and Tuberculosis Research, Ethiopian Public Health Institute, Addis Ababa, Ethiopia

**Keywords:** female sex workers, multinomial logistic regression, HIV vulnerability, public health intervention, urban sex work, cross-sectional study

## Abstract

**Background:**

Understanding the multifactorial drivers of female sex workers' (FSWs) engagement in Ethiopia is essential for designing effective public health interventions. While economic drivers are often emphasized, the roles of family, social, and geographic contexts remain underexplored.

**Methods:**

We analyzed data from a cross-sectional bio-behavioral survey of 6,085 FSWs across 16 Ethiopian urban centers conducted from December 2019 to April 2020. Multinomial logistic regression was used to assess associations between primary motivations for engaging in sex work—categorized as economic, family-related, combined economic-family, or social/behavioral—and socio-demographic, behavioral, and health-related factors.

**Results:**

Economic reasons were most common (41.7%), followed by family-related (22.7%), combined economic-family (21.0%), and social/behavioral (14.6%) motivations. Geographic variation was evident: FSWs in Addis Ababa, the capital in central Ethiopia, had lower odds of reporting family-related reasons (AOR = 0.52) than those in Adama, another central commercial city. FSWs in Dessie–Kombolcha, in northeastern Ethiopia, were more likely to report social or behavioral motivations (AOR = 2.02). Age, education, marital status, income, and healthcare access were also significant predictors. Women aged 35–59 were less likely to cite family (AOR = 0.50) or social motivations (AOR = 0.55), while those with secondary education were more likely to report family-related reasons (AOR = 1.54). Limited healthcare access and early initiation into sex work were associated with non-economic drivers.

**Conclusion:**

FSWs' engagement is influenced by intersecting economic, familial, and geographic factors. Tailored interventions should consider age, location, and service accessibility. The cross-sectional design limits causal interpretation.

## 1 Introduction

Sex work, as defined by the World Health Organization, refers to “the provision of sexual services for money or goods, either regularly or occasionally” (1). In many low-income countries, including Ethiopia, sex work is shaped by intersecting structural factors such as poverty, gender inequality, and limited economic opportunities (1–4). It often serves as a survival strategy for women facing financial hardship, marital instability, or limited access to formal employment (5–7). In Sub-Saharan Africa (SSA), including Ethiopia, socio-cultural pressures, early marriage, educational barriers, and family responsibilities frequently compel women to enter sex work (5, 6, 8, 9). Urban centers present distinct dynamics where economic necessity, particularly among younger women, is a dominant motivator (10–12), while family obligations and psychosocial factors also play important roles (13, 14). The criminalization and stigmatization of sex work further marginalize female sex workers (FSWs), limiting their access to health services and increasing their risk of HIV/AIDS and other health harms (15–17). Beyond SSA, studies from Southeast Asia and Latin America underscore the importance of migration, tourism economies, gender norms, and interpersonal violence in shaping sex work trajectories. In Thailand and Cambodia, for example, the combination of limited employment and peer influence drives early entry into sex work (18). In Brazil and Mexico, domestic violence, early motherhood, and urban precarity are strong determinants of sex work, highlighting intersections between personal trauma and structural disadvantage (19, 20). These insights add critical perspective to the Ethiopian context, where motivations are often interpreted narrowly through an economic lens. Geographic variation significantly influences motivations for sex work in Ethiopia. This study emphasizes major urban centers not only for their geographic coverage but for their strategic relevance. Cities such as Adama, a central commercial hub along the Addis Ababa–Djibouti corridor; Dessie–Kombolcha, located in the northeastern highlands; and Addis Ababa, the centrally located capital, are characterized by high mobility, dense populations of FSWs, and differing socio-economic conditions (10, 11, 21). These cities—located along vital transit routes—present unique cultural and behavioral profiles. Rural-based FSWs often cite family obligations, while those in Addis Ababa are more commonly driven by financial motives (10, 21–23). While financial hardship remains salient—with 41.7% of FSWs nationally citing it as their primary reason (10, 24)—family-related factors and behavioral influences (e.g., peer networks, coercion) also play critical roles (25).

From a theoretical perspective, economic theory frames sex work as a rational labor market response to constrained job options (26), whereas feminist theory underscores how systemic gender inequality channels women into marginal labor sectors (6, 27). Socio-demographic, behavioral, and contextual factors—including age, marital status, educational attainment, income, and healthcare access—all shape sex work entry (6, 27, 28). Psychosocial vulnerabilities such as early trauma, mental health issues, and community stigma further complicate women's experiences (29, 30). These drivers evolve over time, as longer durations in sex work often reflect shifting household needs or lack of exit opportunities (30). Despite a growing body of literature in Ethiopia and SSA, most studies adopt binary logistic or descriptive approaches and often treat motivations for sex work monolithically—typically focusing on HIV vulnerability or economic drivers (10, 15, 24, 28). Few have disaggregated motivations into multiple categories (e.g., economic, family, social and behavioral) or examined how these motivations intersect with geography, education, and healthcare access. Additionally, studies that use regression techniques often assume a dichotomous outcome, limiting explanatory power (25).

**Figure 1:**
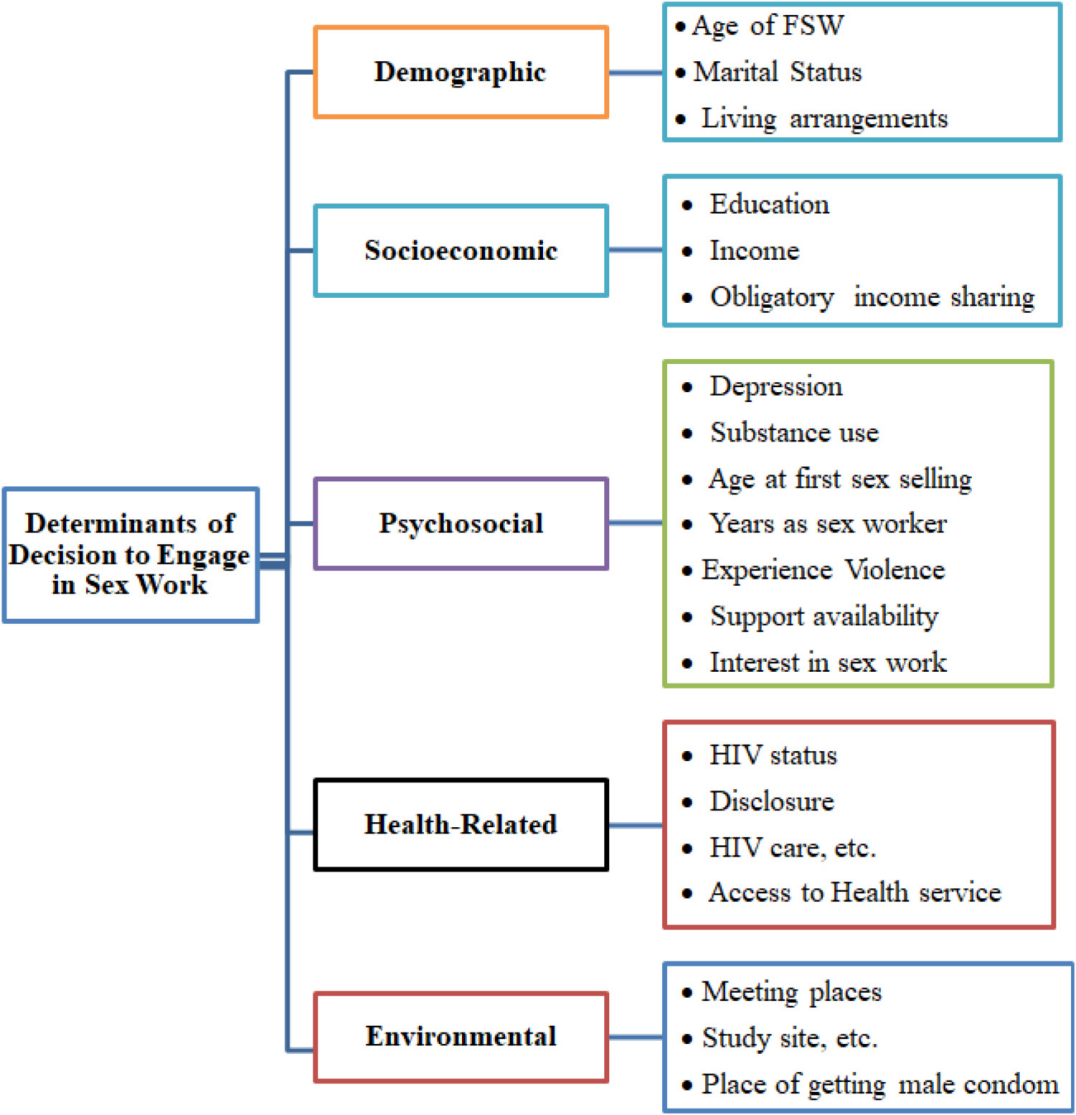
Diagram representing the conceptual framework.

This study addresses these gaps by applying multinomial logistic regression (MLR)—a suitable technique for modeling non-ordinal categorical outcomes—to explore diverse and context-specific motivations for sex work. MLR enables comparison across multiple motivation types while adjusting for demographic, behavioral, and health-related covariates (31). By combining quantitative modeling with rich geographic diversity, this study contributes a more layered understanding of why Ethiopian women engage in sex work, with implications for targeted public health interventions. Figure 1 presents the conceptual framework guiding this study. It outlines how broader socio-demographic, health, and geographic factors influence the core motivations for engaging in sex work—categorized as economic, family-related, and social/behavioral. The framework emphasizes the interconnected nature of these influences, highlighting the importance of addressing economic strain alongside family obligations and psychosocial dynamics in understanding FSWs' decision-making (Figure 1).

## 2 Materials and methods

### 2.1 Study setting and population

According to national estimates, Ethiopia has a substantial population of female sex workers (FSWs), particularly concentrated in urban centers and along major transport routes (7, 10). This study was conducted in 16 cities—including Addis Ababa, Bahir Dar, Dire Dawa, Hawassa, Adama, Arba Minch, Dessie–Kombolcha, Dilla, Gambella, Gonder, Harar, Jimma, and others—which serve as commercial, administrative, and transit hubs. These urban areas are marked by rapid urbanization and high mobility, attracting diverse populations and creating socio-economic conditions that influence sex work engagement. Exploring these varied settings supports a context-specific understanding of the economic, familial, and social drivers of sex work in Ethiopia.

### 2.2 Study design and duration

A cross-sectional bio-behavioral survey was conducted from December 2019–April 2020 across 16 urban sites in Ethiopia. The study aimed to explore the underlying motivations for engagement in sex work among female sex workers (FSWs), alongside relevant socio-demographic and behavioral characteristics.

### 2.3 Target and study population

Female sex workers (FSWs) were defined as biologically female individuals aged 15 years or older who had exchanged sex for money or goods within the past month and had at least four clients in the last 30 days. This definition follows Ethiopia's national bio-behavioral surveillance protocol and aligns with UNAIDS standards (32). Eligible participants were those living or working in the 16 selected cities. Exclusion criteria included living outside the study areas, inability to provide informed consent, or refusal to participate in either the behavioral interview or biological sample collection. A sample refers to blood specimens collected for HIV, hepatitis B, and hepatitis C testing. Respondent-Driven Sampling (RDS) was used for recruitment, starting with 6–10 diverse seeds per site. Seeds were selected to reflect variation in age, work venue, and mobility. Each was given three coupons to recruit peers, enabling access to a hidden population and allowing for RDS-weighted analysis (6, 33, 34).

### 2.4 Sampling and sample size determination

The sample size was estimated using the single population proportion formula, assuming a 95% confidence level, a 5% margin of error, and a 2% prevalence of FSWs in Ethiopia. Adjustments were made for design effects and non-response rates, resulting in a final sample size of 6,085 participants proportionally allocated across the 16 cities based on population size (35).

### 2.5 Sampling procedure

Respondent-driven sampling (RDS) was used for recruitment, beginning with carefully selected seed participants representing different sex work types, age groups, and geographic locations. Each seed received three recruitment coupons to distribute within their social networks. Recruitment continued until RDS equilibrium was reached to ensure a diverse and representative sample. RDS-adjusted weights were applied to minimize bias, and homophily indices and equilibrium diagnostics were assessed (34). An anonymous fingerprint-based coding system was implemented to prevent duplicate enrollments. Recruitment was conducted in hotspot locations such as bars, hotels, streets, and mobile phone networks with the support of local organizations working with FSWs. Key parameters, including HIV status, type of sex work, and condom use, were monitored to track progress toward equilibrium.

### 2.6 Data collection and management

Data collection was conducted using a structured, pre-tested questionnaire developed in English and translated into local languages. The questionnaire covered demographics, socio-economic factors, family responsibilities, and motivations for engaging in sex work. Open Data Kit (ODK) software was used on tablet computers for real-time data collection. Data collectors underwent training on RDS methodology, ethical considerations, and survey administration, and a pilot survey was conducted in Bishoftu to refine the data collection procedures before full implementation.

### 2.7 Study variables

#### 2.7.1 Outcome variable

The primary outcome variable was the self-reported main reason for initiating sex work, classified into four mutually exclusive categories. The first category, economic reasons, included motivations such as the need for better income, unemployment, financial debt, or lack of alternative employment opportunities. The second category, family-related reasons, encompassed factors such as supporting children or other dependents, the death of parents, family conflict, or marital separation. The third category was combined economic and family-related reasons, reflected situations where both financial hardship and family responsibilities were cited. The fourth category, social or behavioral reasons, included peer pressure, being misled or influenced by others, coercion, or being forced into sex work. Peer influence was defined as entry through recruitment or modeling by peers (14), while coercion referred to involuntary engagement. Personal preference was used to denote voluntary choice based on perceived autonomy or job satisfaction, consistent with global classifications (16).

#### 2.7.2 Predictor variables

Predictors were grouped into four domains: demographic (age, marital status, education), socio-economic (monthly income, duration in sex work, income-sharing), behavioral (age at initiation, interest in the work, venue of client meetings), and health-related (HIV status, healthcare access, condom source awareness). These variables were chosen based on theoretical relevance and prior evidence linking them to sex work motivations (27). Some variables, like partner violence or migration, were unavailable in the dataset. Future research could expand on these dimensions.

### 2.8 Statistical analysis

Data collected using Open Data Kit (ODK) were cleaned in Excel and analyzed in R (version 4.3.0). Respondent-Driven Sampling (RDS) procedures—including recruitment tree construction, weight generation, and assumption checks—were performed using the RDS package. Descriptive statistics and chi-square tests assessed associations between categorical variables. Variables with *p*-values < 0.20 in bivariate analysis were included in a multinomial logistic regression to examine associations between motivation types and socio-demographic factors. This method was preferred for its suitability with unordered categorical outcomes. The model accounted for clustering within RDS networks, and results were reported as adjusted odds ratios (AORs) with 95% confidence intervals. Variables unrelated to the study objective, such as hepatitis B and C status, were excluded from the analysis. We assessed multicollinearity using variance inflation factors (VIFs) and evaluated model fit with McFadden's pseudo R² and likelihood ratio tests (33, 36).

### 2.9 Ethical considerations

The study received ethical approval from the Scientific and Ethical Research Office (SERO) of the Ethiopian Public Health Institute. Informed consent was obtained in participants' preferred language. For minors aged 15–17, an IRB-approved waiver of parental consent was used in line with international guidelines to protect adolescent autonomy. Given the stigma around sex work, strong confidentiality safeguards were in place. No names were recorded, interviews were private, and data were encrypted and de-identified. To prevent repeat participation, a fingerprint-based code system (non-identifiable and encrypted) was used with prior ethical approval and community sensitization. Participation was voluntary, and those declining fingerprinting were not excluded. Participants received compensation, and those testing positive for HIV or hepatitis were referred to care. The study adhered to the Declaration of Helsinki and national guidelines for research involving human participants.

## 3 Results

### 3.1 Overview of study population characteristics

Table 1 shows the distribution of primary motivations for sex work among 6,085 FSWs from 16 diverse urban sites in Ethiopia, serving as a basis for subsequent analyses. The highest representation came from Addis Ababa (18.1%), the centrally located capital and largest city in Ethiopia. Adama (11.1%), a major commercial hub situated southeast of the capital along the Addis Ababa–Djibouti transport corridor, also contributed significantly. Other key sites included Hawassa (8.6%), the capital of the Sidama region in southern Ethiopia; and Dire Dawa (7.1%), a chartered city in the eastern part of the country with high mobility and trade activity. Additional contributions came from Gambella (7.7%), located in the western lowlands near the South Sudan border; Jimma (4.2%) and Mizan Aman (4.2%) in the southwest; Nekemte (4.2%) in the western highlands; and other cities such as Arba Minch, Bahir Dar, Dessie/Kombolcha, Dilla, Logia/Semera, Shashemane, Harar, and Gondar (each contributing approximately 4%). Regarding motivations for entering sex work, economic reasons were most commonly cited (41.7%, *n* = 2,539), including income needs and a lack of alternative employment. Family-related factors—such as the need to support children, divorce, or household conflict—were reported by 22.7% (*n* = 1,382) of participants. Additionally, 21.0% (*n* = 1,278) indicated a combination of economic and family-related pressures, while 14.6% (*n* = 886) cited social or behavioral factors, including peer influence, coercion, or personal preference.

**Table 1:** Distribution of FSWs' primary motivations for engaging in sex work by study site (*N* = 6,085).

Study site (Location)	Reason for engaging in sex work				Total
	Economic Reasons	Family Issues	Economic + Family	Others	
Adama	239 (35.4%)	232 (34.3%)	123 (18.2%)	82 (12.1%)	676 (11.1%)
Addis Ababa	530 (48.1%)	235 (21.3%)	161 (14.6%)	175 (15.9%)	1,101 (18.1%)
Arba Minch	99 (39.4%)	64 (25.5%)	57 (22.7%)	31 (12.4%)	251 (4.1%)
Bahir Dar	170 (45.7%)	54 (14.5%)	82 (22%)	66 (17.7%)	372 (6.1%)
Dessie/Kombolcha	88 (35.1%)	57 (22.7%)	46 (18.3%)	60 (23.9%)	251 (4.1%)
Dilla	99 (39.4%)	43 (17.1%)	81 (32.3%)	28 (11.2%)	251 (4.1%)
Diredawa	171 (39.4%)	57 (13.1%)	173 (39.9%)	33 (7.6%)	434 (7.1%)
Gamebella	212 (45.3%)	107 (22.9%)	110 (23.5%)	39 (8.3%)	468 (7.7%)
Gonder	134 (53.6%)	35 (14%)	68 (27.2%)	13 (5.2%)	250 (4.1%)
Harar	112 (46.3%)	51 (21.1%)	59 (24.4%)	20 (8.3%)	242 (4%)
Hawassa	165 (31.6%)	180 (34.5%)	66 (12.6%)	111 (21.3%)	522 (8.6%)
Jimma	131 (51.6%)	46 (18.1%)	58 (22.8%)	19 (7.5%)	254 (4.2%)
Logia/Semera	102 (40.6%)	39 (15.5%)	48 (19.1%)	62 (24.7%)	251 (4.1%)
Mizan	116 (45.5%)	40 (15.7%)	56 (22%)	43 (16.9%)	255 (4.2%)
Nekemte	85 (33.1%)	67 (26.1%)	44 (17.1%)	61 (23.7%)	257 (4.2%)
Shashemane	86 (34.4%)	75 (30%)	46 (18.4%)	43 (17.2%)	250 (4.1%)
Total	2,539 (41.7%)	1,382 (22.7%)	1,278 (21%)	886 (14.6%)	6,085 (100%)

As shown in Table 1, the distribution of motivations varied across cities, suggesting that geographic location—and the associated socio-economic environment—may influence the primary reasons why women enter sex work. These descriptive findings provide important contextual background for interpreting the multivariable analysis presented in later sections.

### 3.2 Socio-demographic characteristics of FSWs

Table 2 presents the distribution of primary motivations for engaging in sex work across socio-demographic characteristics among 6,085 female sex workers (FSWs) in Ethiopia. Economic motivations were most common overall (41.7%), followed by family-related (22.7%), combined economic and family (21.0%), and social or behavioral motivations (14.6%). Age was strongly associated with motivational differences. Among FSWs aged 15–19 years, 40.5% cited economic reasons and 25.2% family-related, while among those aged 35–59, 49.7% reported economic reasons and only 7.3% social motivations. Motivation also varied by education: FSWs with non-formal education most frequently reported economic reasons (44.9%), while those with secondary or post-secondary education had more varied motivations, including higher family-related (26.2%) and social reasons (13.2%). Marital status revealed distinct patterns. Never-partnered women more often cited economic motivations (44.9%) and social reasons (20.3%), while previously partnered women reported a higher share of combined motivations (31.2%). Among those currently partnered, motivations were more evenly distributed. Income-sharing practices influenced motivations. FSWs who did not share income with others were more likely to report economic motivations (42.3%). In contrast, those sharing with workplace owners or intermediaries reported higher proportions of family-related (up to 27.2%) and social motivations (up to 22.2%). Monthly income also showed a clear gradient. FSWs earning ≤1,500 ETB had the highest proportion reporting economic motivations (46.4%), while those earning >10,000 ETB were more likely to report family-related (30.6%) or social motivations (19.2%).

**Table 2:** Bivariate analysis of socio-demographics by sex work motivation (*N* = 6,085).

Predictor Variables	Categories	Reason for engaging in sex work				Total
		Economic	Family	Econ. + Fam.	Others	
Age of FSW	15–19 yrs.	249 (40.5%)	155 (25.2%)	86 (14%)	125 (20.3%)	615 (10.1%)
	20–24 yrs.	785 (39.6%)	498 (25.2%)	312 (15.8%)	385 (19.4%)	1,980 (32.5%)
	25–29 yrs.	728 (40.1%)	423 (23.3%)	423 (23.3%)	241 (13.3%)	1,815 (29.8%)
	30–34 yrs.	370 (43.2%)	177 (20.7%)	234 (27.3%)	75 (8.8%)	856 (14.1%)
	35–59 yrs.	407 (49.7%)	129 (15.8%)	223 (27.2%)	60 (7.3%)	819 (13.5%)
Highest grade attended	Non-formal Education	471 (44.9%)	183 (17.5%)	238 (22.7%)	156 (14.9%)	1,048 (17.2%)
	Certificate and above	62 (54.9%)	18 (15.9%)	22 (19.5%)	11 (9.7%)	113 (1.9%)
	Primary 1st cycle	328 (38.6%)	188 (22.1%)	193 (22.7%)	141 (16.6%)	850 (14%)
	Primary 2nd cycle	1,112 (41%)	637 (23.5%)	565 (20.8%)	399 (14.7%)	2,713 (44.6%)
	Secondary school	566 (41.6%)	356 (26.2%)	260 (19.1%)	179 (13.2%)	1,361 (22.4%)
Marital status	Currently Partnered	109 (47.2%)	43 (18.6%)	37 (16%)	42 (18.2%)	231 (3.8%)
	Previously Partnered	1,108 (38.1%)	646 (22.2%)	908 (31.2%)	247 (8.5%)	2,909 (47.8%)
	Never Partnered	1,322 (44.9%)	693 (23.5%)	333 (11.3%)	597 (20.3%)	2,945 (48.4%)
Obligatory sex income sharing with	None	2,290 (42.3%)	1,212 (22.4%)	1,152 (21.3%)	756 (14%)	5,410 (88.9%)
	Owner of Workplace	156 (33.6%)	126 (27.2%)	79 (17%)	103 (22.2%)	464 (7.6%)
	Middle Person	52 (40.3%)	26 (20.2%)	31 (24%)	20 (15.5%)	129 (2.1%)
	Others	41 (50%)	18 (22%)	16 (19.5%)	7 (8.5%)	82 (1.3%)
Living with sexual partner	Yes	206 (41.4%)	105 (21.1%)	106 (21.3%)	81 (16.3%)	498 (8.2%)
	No	2,333 (41.8%)	1,277 (22.9%)	1,172 (21%)	805 (14.4%)	5,587 (91.8%)
Average monthly income from selling sex	<=1,500 ETB	417 (46.4%)	179 (19.9%)	188 (20.9%)	114 (12.7%)	898 (14.8%)
	1,501–3,000 ETB	814 (42.4%)	402 (20.9%)	429 (22.3%)	275 (14.3%)	1,920 (31.6%)
	3,001–5,000 ETB	631 (42.7%)	341 (23.1%)	309 (20.9%)	197 (13.3%)	1,478 (24.3%)
	5,001–7,500 ETB	300 (39.7%)	183 (24.2%)	163 (21.6%)	110 (14.6%)	756 (12.4%)
	7,501–10,000 ETB	267 (38.7%)	172 (24.9%)	127 (18.4%)	124 (18%)	690 (11.3%)
	>10,000 ETB	110 (32.1%)	105 (30.6%)	62 (18.1%)	66 (19.2%)	343 (5.6%)
Total		2,539 (41.7%)	1,382 (22.7%)	1,278 (21%)	886 (14.6%)	6,085 (100%)

These patterns provide contextual insight into the socio-demographic variation in motivations for engaging in sex work, supporting interpretation of adjusted results in subsequent analyses.

### 3.3 Age and income-related trends in motivations for engaging in sex work

**Figure 2:**
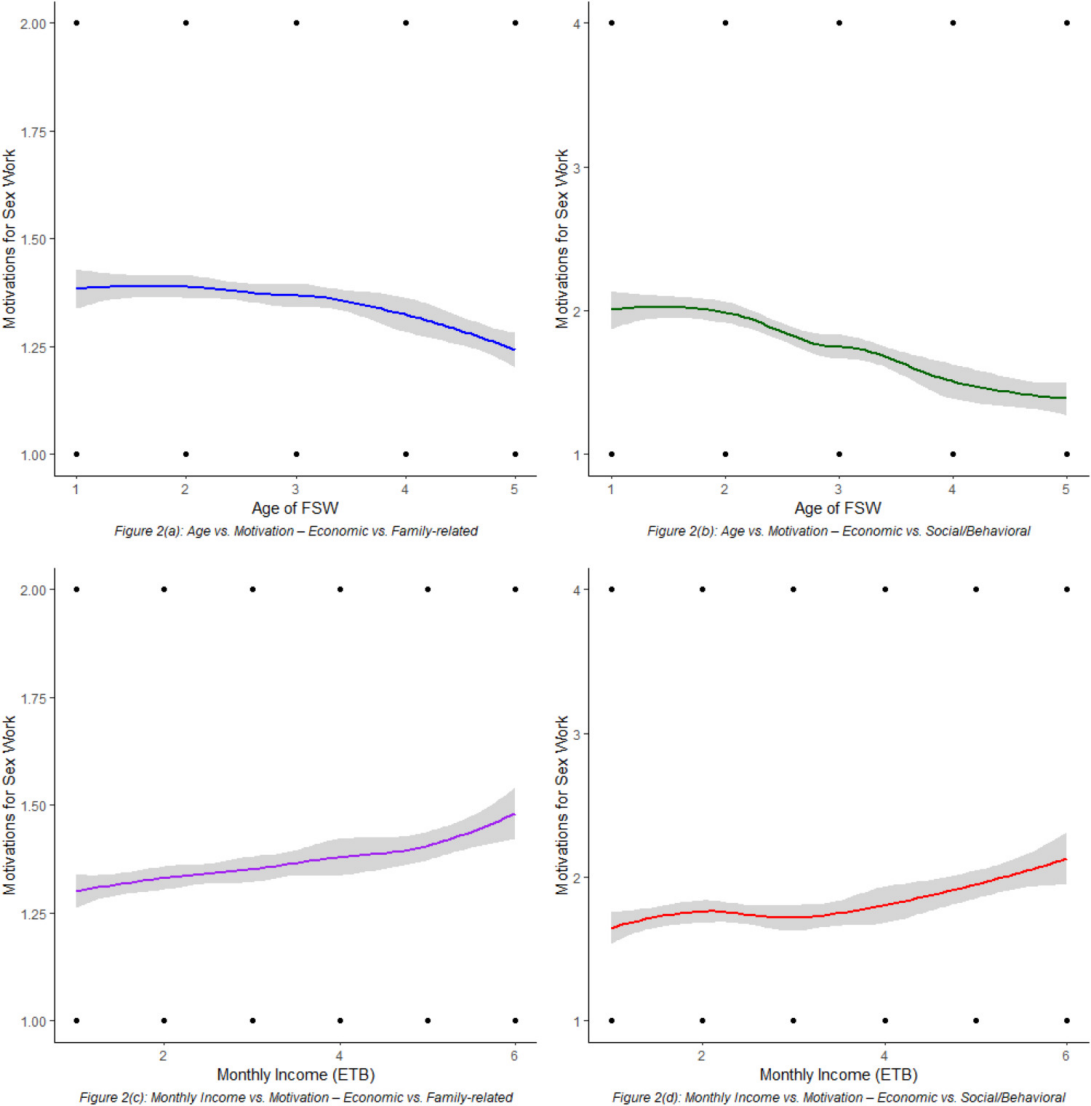
LOESS-smoothed trends showing associations between age, income, and primary motivations for engaging in sex work among FSWs, with 95% confidence intervals.

As illustrated in Figure 2, motivations for engaging in sex work varied by both age and income level, with 95% confidence intervals indicating the precision of the estimates. Younger female sex workers (FSWs) were more likely to report family-related or social/behavioral motivations compared to economic ones, while older FSWs showed a gradual shift toward citing economic necessity. Similarly, higher income levels were modestly associated with increased reporting of family-related or social motivations over purely economic reasons. These trends suggest that motivations for sex work are influenced not only by financial hardship but also by evolving social, familial, and contextual factors across the life course (Figure 2).

While age and income offer important insights into FSWs' motivations, a fuller understanding requires examining behavioral and health-related factors. The next section explores how personal behaviors, health status, and service access relate to motivations for engaging in sex work.

### 3.4 Behavioral and health-related characteristics of FSWs

Table 3 presents the distribution of behavioral and health-related characteristics of female sex workers (FSWs) by their primary motivation for engaging in sex work. Motivation types varied across levels of interest in sex work. Among those who reported always enjoying sex work, 48.8% cited economic reasons, compared to 41.7% of those who never enjoyed it. In contrast, 17.2% of the latter group reported social or behavioral reasons, suggesting lower interest was linked to non-economic motivations.

**Table 3:** Bivariate distribution of behavioral and health characteristics by sex work motivation (*N* = 6,085).

Predictor Variables	Categories	Reason for engaging in sex work				
		Economic	Family	Econ. + Fam.	Others	Total
Interest or pleasure in selling sex	Not at all (never)	1,008 (41.7%)	532 (22%)	460 (19%)	416 (17.2%)	2,416 (39.7%)
	Sometimes	1,426 (41.3%)	817 (23.7%)	767 (22.2%)	444 (12.9%)	3,454 (56.8%)
	All the time	105 (48.8%)	33 (15.3%)	51 (23.7%)	26 (12.1%)	215 (3.5%)
Age at first sex selling	<20 yrs.	883 (37.9%)	613 (26.3%)	377 (16.2%)	456 (19.6%)	2,329 (38.3%)
	20–24 yrs.	974 (41.5%)	528 (22.5%)	509 (21.7%)	338 (14.4%)	2,349 (38.6%)
	25+ yrs.	682 (48.5%)	241 (17.1%)	392 (27.9%)	92 (6.5%)	1,407 (23.1%)
Number of years as sex worker	<=1	520 (44.3%)	252 (21.4%)	196 (16.7%)	207 (17.6%)	1,175 (19.3%)
	2–4	871 (41.6%)	493 (23.6%)	417 (19.9%)	312 (14.9%)	2,093 (34.4%)
	5–7	751 (40.6%)	422 (22.8%)	433 (23.4%)	244 (13.2%)	1,850 (30.4%)
	8+	397 (41.1%)	215 (22.2%)	232 (24%)	123 (12.7%)	967 (15.9%)
Usual place of meeting clients	Commercial Places	1173 (40.1%)	683 (23.4%)	603 (20.6%)	466 (15.9%)	2925 (48.1%)
	Public Spaces	1,022 (42.8%)	555 (23.3%)	490 (20.5%)	320 (13.4%)	2,387 (39.2%)
	Private Settings	221 (44.2%)	81 (16.2%)	140 (28%)	58 (11.6%)	500 (8.2%)
	Others	123 (45.1%)	63 (23.1%)	45 (16.5%)	42 (15.4%)	273 (4.5%)
HIV Status	Negative	2,062 (41.6%)	1,135 (22.9%)	1,011 (20.4%)	747 (15.1%)	4,955 (81.4%)
	Positive	477 (42.2%)	247 (21.9%)	267 (23.6%)	139 (12.3%)	1,130 (18.6%)
Health care services	Yes	1,667 (43.3%)	794 (20.6%)	925 (24%)	464 (12.1%)	3,850 (63.3%)
	No	872 (39%)	588 (26.3%)	353 (15.8%)	422 (18.9%)	2,235 (36.7%)
Awareness about place to get male condoms	Health Facilities	622 (40.1%)	353 (22.8%)	330 (21.3%)	246 (15.9%)	1,551 (25.5%)
	Commercial Sources	1,272 (42.5%)	703 (23.5%)	598 (20%)	421 (14.1%)	2,994 (49.2%)
	Non-Commercial	419 (40.4%)	204 (19.7%)	261 (25.2%)	152 (14.7%)	1,036 (17%)
	Unknown	226 (44.8%)	122 (24.2%)	89 (17.7%)	67 (13.3%)	504 (8.3%)
Total		2,539 (41.7%)	1,382 (22.7%)	1,278 (21%)	886 (14.6%)	6,085 (100%)

Age at first involvement showed similar variation. Among FSWs who entered the profession at age 25 or older, 48.5% reported economic reasons and only 6.5% cited social motivations. In contrast, among those who started before age 20, economic (37.9%), family (26.3%), and social (19.6%) motivations were more evenly distributed. Time in sex work followed a parallel trend. Among newer entrants (≤1 year), 44.3% cited economic reasons and 17.6% social reasons. With longer durations, the proportion citing economic motivations declined slightly, and combined motivations increased (e.g., 24.0% among those with 8+ years). Meeting location was also associated with motivational type. FSWs operating in commercial places cited economic reasons most often (40.1%), while those in private settings showed a higher percentage reporting combined motivations (28.0%). Regarding health-related characteristics, 42.2% of HIV-positive FSWs reported economic motivations and 23.6% reported combined reasons. FSWs with access to healthcare services were more likely to cite economic (43.3%) or combined motivations (24.0%) than those without access, who more often cited family (26.3%) or social reasons (18.9%). Motivations also varied by condom source awareness. Those relying on non-commercial or unknown sources were more likely to cite combined motivations (up to 25.2%) than those using health facilities or commercial sources.

These findings highlight the influence of behavioral, health, and environmental factors on sex work motivations, providing important context for tailored intervention strategies.

### 3.5 Patterns and predictors of engagement in female sex work

Table 4 presents the results of the multinomial logistic regression analysis examining factors associated with the reasons FSWs engage in sex work, categorized as family-related, economic combined with family, and other social/behavioral reasons (see Table 4). FSWs from Addis Ababa, Ethiopia's capital, were significantly less likely than those from Adama to report family-related [AOR = 0.52, 95% CI (0.40, 0.67), *p* < .001] or combined reasons [AOR = 0.72, 95% CI (0.53, 0.97), *p* = .03] relative to economic motivations. Similarly, those from Bahir Dar, the northern Amhara regional capital, were 60% less likely to cite family reasons [AOR = 0.40, 95% CI (0.27, 0.59), *p* < .001]. FSWs from Dilla (Southern Nations), Diredawa (eastern Ethiopia), and Gonder (northwest Amhara) also showed lower odds of family-related motivations compared to economic reasons (AORs 0.32–0.53, all *p* < .001). In contrast, FSWs from Dilla and Dire Dawa—major urban centers in southern and eastern Ethiopia—were significantly more likely than those from Adama to report combined economic and family reasons over economic reasons alone [Dilla: AOR = 1.90, 95% CI (1.28, 2.81); Dire Dawa: AOR = 1.86, 95% CI (1.33, 2.59)]; both *p* < .001]. FSWs from smaller or emerging towns in northern and western Ethiopia, such as Dessie—Kombolcha and Nekemte, had significantly higher odds of citing social or behavioral (“other”) motivations rather than economic ones (Dessie—Kombolcha: AOR = 2.02; Nekemte: AOR = 2.61; both *p* < .001). Elevated odds of reporting “other” motivations were also observed among FSWs in Hawassa, Semera—Logia (Afar), and Shashemene, with Semera—Logia showing the highest increase [AOR = 2.40, 95% CI (1.54, 3.74), *p* < .001]. Age also significantly predicted motivational patterns. Compared to FSWs aged 15–19, those aged 35–59 were less likely to report family-related motivations [AOR = 0.50, 95% CI (0.31, 0.81), *p* = .00] or social/behavioral motivations [AOR = 0.55, 95% CI (0.31, 0.97), *p* = .04], relative to economic motivations. The 30–34 age group also showed reduced odds of social/behavioral motivations, although the association was not statistically significant [AOR = 0.67, 95% CI (0.41, 1.11), *p* = .12].

**Table 4:** Predictors influencing FSWs' engagement in Sex work.

Predictor Variables	Reasons for Selling Sex								
	Family Issues			Economic + Family			Others		
	AOR	p.value	95% CI	AOR	p.value	95% CI	AOR	p.value	95% CI
(Intercept)	0.53	0.03	(0.30, 0.95)	0.22	0.00	(0.12, 0.40)	0.44	0.01	(0.23, 0.85)
Study site [Reference: Adama]									
Addis Ababa	0.52	0.00	(0.40, 0.67)	0.72	0.03	(0.53, 0.97)	1.09	0.62	(0.78, 1.50)
Arba Minch	0.75	0.15	(0.50, 1.12)	1.43	0.11	(0.92, 2.21)	1.19	0.52	(0.70, 2.01)
Bahir Dar	0.40	0.00	(0.27, 0.59)	1.21	0.31	(0.83, 1.76)	1.32	0.19	(0.87, 2.00)
Dessie–Kombolcha	0.65	0.03	(0.44, 0.97)	1.02	0.92	(0.66, 1.58)	2.02	0.00	(1.31, 3.13)
Dilla	0.40	0.00	(0.26, 0.61)	1.90	0.00	(1.28, 2.81)	0.71	0.19	(0.42, 1.18)
Diredawa	0.40	0.00	(0.28, 0.58)	1.86	0.00	(1.33, 2.59)	0.79	0.32	(0.49, 1.27)
Gamebella	0.53	0.00	(0.39, 0.74)	1.15	0.43	(0.81, 1.62)	0.59	0.02	(0.38, 0.93)
Gonder	0.32	0.00	(0.21, 0.49)	1.25	0.26	(0.85, 1.86)	0.34	0.00	(0.18, 0.64)
Harar	0.53	0.00	(0.36, 0.79)	0.96	0.83	(0.64, 1.43)	0.64	0.12	(0.36, 1.12)
Hawassa	1.20	0.23	(0.89, 1.61)	0.91	0.62	(0.62, 1.33)	2.05	0.00	(1.41, 2.99)
Jimma	0.42	0.00	(0.28, 0.63)	0.89	0.56	(0.60, 1.32)	0.55	0.04	(0.31, 0.97)
Logia	0.48	0.00	(0.31, 0.74)	1.13	0.57	(0.73, 1.75)	2.40	0.00	(1.54, 3.74)
Mizan	0.37	0.00	(0.24, 0.57)	1.24	0.34	(0.80, 1.91)	1.23	0.40	(0.76, 2.00)
Nekemite	0.96	0.84	(0.65, 1.43)	1.34	0.21	(0.85, 2.11)	2.61	0.00	(1.65, 4.11)
Shashemane	1.04	0.84	(0.71, 1.52)	1.25	0.32	(0.80, 1.96)	1.75	0.02	(1.09, 2.82)
Age of FSW [Reference: 15–19 years]									
20–24 years	0.98	0.89	(0.76, 1.27)	0.87	0.37	(0.64, 1.18)	1.09	0.56	(0.82, 1.44)
25–29 years	0.90	0.52	(0.65, 1.25)	0.90	0.58	(0.62, 1.30)	0.87	0.48	(0.60, 1.27)
30–34 years	0.77	0.21	(0.50, 1.17)	0.83	0.42	(0.53, 1.30)	0.67	0.12	(0.41, 1.11)
35–59 years	0.50	0.00	(0.31, 0.81)	0.67	0.11	(0.41, 1.10)	0.55	0.04	(0.31, 0.97)
Education [Reference: Non-formal Education]									
Certificate and above	0.65	0.14	(0.37, 1.15)	0.99	0.97	(0.58, 1.69)	0.35	0.00	(0.18, 0.71)
Primary 1st cycle	1.31	0.04	(1.02, 1.70)	1.24	0.09	(0.97, 1.59)	1.06	0.70	(0.80, 1.40)
Primary 2nd cycle	1.32	0.01	(1.07, 1.63)	1.21	0.06	(0.99, 1.48)	0.79	0.05	(0.63, 1.00)
Secondary school	1.54	0.00	(1.22, 1.93)	1.19	0.14	(0.94, 1.50)	0.68	0.00	(0.52, 0.89)
Marital Status [Reference: Currently Partnered]									
Previously Partnered	1.76	0.00	(1.20, 2.58)	2.57	0.00	(1.73, 3.81)	0.64	0.03	(0.43, 0.96)
Never Partnered	1.07	0.71	(0.74, 1.57)	0.76	0.17	(0.51, 1.13)	0.85	0.40	(0.57, 1.25)
Monthly income from Sex Work [Reference: <=1,500 ETB]									
1,501–3,000 ETB	1.13	0.28	(0.90, 1.42)	1.20	0.11	(0.96, 1.50)	1.34	0.03	(1.03, 1.74)
3,001–5,000 ETB	1.12	0.34	(0.88, 1.43)	1.24	0.08	(0.97, 1.59)	1.21	0.20	(0.90, 1.61)
5,001–7,500 ETB	1.19	0.22	(0.90, 1.58)	1.46	0.01	(1.10, 1.94)	1.47	0.02	(1.06, 2.06)
7,501–10,000 ETB	1.15	0.36	(0.86, 1.53)	1.33	0.07	(0.98, 1.81)	1.72	0.00	(1.22, 2.41)
>10,000 ETB	1.56	0.02	(1.09, 2.23)	1.57	0.03	(1.06, 2.34)	2.39	0.00	(1.57, 3.63)
Age at First Sex initiation [Reference: <=20 yrs.]									
20–24 yrs.	0.77	0.01	(0.64, 0.93)	0.98	0.86	(0.80, 1.21)	0.78	0.02	(0.63, 0.97)
>=25	0.63	0.00	(0.47, 0.86)	0.95	0.71	(0.70, 1.28)	0.48	0.00	(0.32, 0.70)
Duration in selling sex [Reference: <=1 years]									
2–4 years	1.15	0.16	(0.95, 1.41)	1.15	0.21	(0.93, 1.42)	1.03	0.81	(0.82, 1.29)
5–7 years	1.19	0.14	(0.94, 1.49)	1.28	0.04	(1.01, 1.63)	1.09	0.50	(0.84, 1.42)
>=8 years	1.39	0.05	(1.00, 1.92)	1.28	0.14	(0.92, 1.77)	1.39	0.10	(0.94, 2.05)
Condom access awareness [Reference: Health Facilities]									
Commercial	0.86	0.09	(0.73, 1.02)	0.97	0.72	(0.81, 1.15)	0.80	0.03	(0.66, 0.97)
Non-Commercial	0.88	0.25	(0.70, 1.09)	1.14	0.23	(0.92, 1.42)	0.96	0.78	(0.75, 1.24)
Unknown	1.02	0.91	(0.78, 1.32)	0.79	0.12	(0.59, 1.06)	0.77	0.12	(0.56, 1.07)
Healthcare access [Reference: Yes]									
Healthcare access No	1.41	0.00	(1.22, 1.63)	0.92	0.29	(0.78, 1.07)	1.52	0.00	(1.29, 1.79)

All predictors were tested for multicollinearity using VIFs and found to be acceptable (VIF < 2.5). Model fit assessed using McFadden pseudo R² = 0.18.

Education showed mixed effects on motivations for engaging in sex work. Compared to FSWs with no formal education, those with secondary education were significantly more likely to report family-related reasons [AOR = 1.54, 95% CI (1.22, 1.93), *p* < .001], but less likely to cite social or behavioral motivations [AOR = 0.68, 95% CI (0.52, 0.89), *p* = .00]. Similarly, FSWs with primary second cycle (grades 5–8) and first cycle (grades 1–4) education were also more likely to report family-related reasons (AOR = 1.32 and 1.31, respectively). Marital status was strongly associated with motivational patterns. Previously partnered FSWs (divorced, separated, or widowed) were significantly more likely than currently partnered women to report family-related reasons [AOR = 1.76, 95% CI (1.20, 2.58), *p* = .00] and combined economic and family motivations [AOR = 2.57, 95% CI (1.73, 3.81), *p* = .00]. However, they were less likely to cite social or behavioral reasons [AOR = 0.64, 95% CI (0.43, 0.96), *p* = .03]. FSWs who had never been partnered showed no significant differences from those currently partnered. Higher monthly income was associated with increased odds of reporting non-economic motivations. FSWs earning more than 10,000 ETB per month were more likely to cite family-related (AOR = 1.56), combined (AOR = 1.57), and social or behavioral reasons (AOR = 2.39) compared to those reporting economic reasons (all *p* < .05). Similarly, those earning 5,001–7,500 ETB had higher odds of reporting combined (AOR = 1.46) and social/behavioral (AOR = 1.47) reasons compared to the lowest income group (≤1,500 ETB). FSWs earning 7,501–10,000 ETB per month were 72% more likely to report social and behavioral motivations [AOR = 1.72, 95% CI (1.22, 2.41), *p* = .00], suggesting that middle- to upper-income earners may have more complex or multidimensional motivations.

Age at initiation also influenced motivations. Compared to FSWs who began sex work at age ≤20, those initiating at age 25 or older were less likely to report family-related (AOR = 0.63) and social or behavioral reasons (AOR = 0.48). Those who started between ages 20–24 also had reduced odds of reporting family-related (AOR = 0.77) and social or behavioral motivations (AOR = 0.78). Duration of engagement in sex work was another significant factor. FSWs with 8 or more years of experience were 39% more likely to cite family-related reasons (AOR = 1.39, *p* = .05), while those with 5–7 years were 28% more likely to report combined economic and family motivations (AOR = 1.28, *p* = .04). These findings suggest that longer involvement in sex work may reflect increasingly complex or evolving life circumstances beyond economic need. FSWs obtaining condoms from commercial sources were 20% less likely to report social or behavioral motivations compared to those accessing condoms through health facilities (AOR = 0.80, *p* = .03). No significant differences were observed among those using non-commercial sources, suggesting that commercial access may provide a form of protective autonomy. Lack of access to healthcare was strongly associated with non-economic motivations: FSWs without access to health services were 41% more likely to cite family-related reasons (AOR = 1.41, *p* < .001) and 52% more likely to report social or behavioral factors (AOR = 1.52, *p* < .001) than those with healthcare access.

Overall, these findings underscore the complex and multifaceted motivations behind FSWs' engagement in sex work. While economic need remains central, many also cite family-related and social or behavioral reasons—shaped by factors such as income, age, education, duration in sex work, condom access, healthcare, and geographic region. Non-economic motivations were more commonly reported in southern, eastern, and pastoral areas, and less so in major urban centers and northern regions. These patterns emphasize the need for tailored, context-specific interventions that address the diverse realities of FSWs across Ethiopia.

## 4 Discussion

This study provides a nuanced, multisite analysis of motivations behind Ethiopian female sex workers' (FSWs) engagement in sex work, using multinomial logistic regression on a large, geographically diverse sample. Economic hardship was the most frequently cited motivation (41.7%), consistent with previous research linking poverty and unemployment to sex work participation (10, 12, 22). Nonetheless, family-related pressures (22.7%), combined economic and familial obligations (21.0%), and social or behavioral influences (14.6%) were also prominent, highlighting the multifactorial nature of motivations that require tailored intervention approaches.

Our analysis revealed important demographic and geographic variation. Younger FSWs (15–24 years) were more likely to report family and social motivations, whereas older women (35–59 years) predominantly cited economic reasons, in line with life course theory perspectives on shifting needs and responsibilities (11, 28). Women with secondary education reported family-related motivations more frequently, suggesting that educational attainment may increase perceived familial obligations without fully mitigating vulnerability (8). Geographic differences were pronounced: FSWs in Addis Ababa, a relatively opportunity-rich urban center, were less likely to cite family reasons, while towns such as Dilla and Dire Dawa—characterized by tighter community structures and limited employment options—had higher reports of family-driven motivations. Cities situated along major trade and migration corridors (Adama, Hawassa, Shashemane) showed distinct motivation patterns, likely influenced by mobility and transactional economies (5, 21). These findings underscore the inadequacy of uniform interventions and the need for region-specific programming.

Different motivational profiles suggest specific policy priorities. Economic hardship-driven FSWs may benefit most from economic empowerment initiatives, such as vocational training, stable employment, and microfinance programs. For those motivated by family pressures, social protection interventions—including childcare support, legal aid for single mothers, and community-based assistance—may mitigate familial burdens. FSWs influenced by social or behavioral factors, particularly younger women, could gain from psychosocial support services like peer mentorship, trauma-informed counseling, and behavioral risk reduction. The observed geographic variation further necessitates place-based approaches, such as harm reduction and mobility-integrated services in transit cities, alongside employment-centered programs in urban hubs.

Several methodological limitations warrant consideration. Respondent-Driven Sampling (RDS), though appropriate for hidden populations, may introduce network biases by over-representing socially connected individuals and underrepresenting isolated or recently engaged FSWs, affecting representativeness. The cross-sectional design prevents causal inference regarding the temporal order of motivations and sex work entry. Moreover, motivations are dynamic and likely evolve over time, but this study captures only a single snapshot. Self-reported data on sensitive topics may be subject to social desirability bias. These limitations highlight the need for longitudinal and mixed-methods research to capture the evolving nature of sex work motivations and explore how broader structural factors, such as housing stability or legal protections, shape vulnerabilities.

## 5 Strengths and limitations

This study's major strength lies in its inclusion of high-risk, often underrepresented female sex workers (FSWs) across 16 diverse urban settings in Ethiopia. The use of respondent-driven sampling (RDS) enabled access to hidden populations and enhanced the geographic relevance of findings for public health planning. However, limitations include potential sampling bias from RDS, as recruitment equilibrium across key variables was not formally assessed. Self-reported measures, including HIV status and motivation, may be influenced by recall or social desirability bias. The cross-sectional design also precludes causal interpretation between predictors and reported motivations. Additionally, the motivational categories, though informed by prior literature and pretesting, may oversimplify complex decision-making processes. The urban focus may limit generalizability to rural or home-based FSWs, and logistical challenges such as inconsistent street naming affected recruitment in some sites. Resource constraints also limited confirmatory testing for health indicators. Despite these limitations, the study offers important insights into the multifactorial drivers of sex work engagement. Future research should incorporate longitudinal or qualitative approaches to strengthen causal inference and contextual understanding.

## 6 Conclusion

This study highlights diverse motivations for sex work in Ethiopia, with economic need, family pressure, and social factors varying by region. Scalable interventions should prioritize financial support and vocational training for economically driven cases, while psychosocial services and community programs are better suited for socially or family-motivated groups. Improving healthcare access remains essential across all contexts. Future qualitative research should explore the roles of stigma, family dynamics, and peer influence using structured frameworks to guide context-specific, gender-sensitive policies.

## Data Availability

The raw data supporting the conclusions of this article will be made available by the authors, without undue reservation.

## Author's note

This research was conducted as part of an ongoing study on the socio-demographic and economic factors influencing female sex workers' engagement in Ethiopia. The authors declare no conflicts of interest and confirm that the study adhered to ethical guidelines and was approved by the appropriate review boards. Data collection was carried out with informed consent from all participants, ensuring their anonymity and confidentiality. For correspondence regarding this article, please contact: DD Ethiopian Public Health Institute, Addis Ababa, Ethiopia, Phone: +251 920 405 359.
